# The future of medicine in the 21^st^ century

**DOI:** 10.4103/2152-7806.70851

**Published:** 2010-10-06

**Authors:** James I. Ausman

**Affiliations:** Editor-in-Chief, Surgical Neurology International, USA

According to futurists Alvin and Heidi Toffler, waves of change are occurring all over the world.[7,17] Some people are still living in the primitive hunter–gatherer civilizations, while others are going through the agricultural wave of change and still some others are in their industrial age, with a few moving into the knowledge age or the information age. In some countries, several of these waves of change are occurring simultaneously, making communication and understanding among the people in these different waves of change difficult. The different waves of change sweeping the world in which people exist under different philosophical and governing systems explain why there will be conflicts and wars for generations to come.

According to futurists, in science, the rate of change and accumulation of new information is asymptotic and will change 1000X faster in the 21st century than it did in the last 5 years of the 20th century.[14] Not only is there a huge amount of knowledge being created but there is also a need to make sense of all of this information. This transformation between waves of change is a transition societies are going through now all over the world. The rapid growth of knowledge will force specialization and interdisciplinary cooperation not only in medicine, but also in other fields because a single person cannot comprehend or know all this information.[1,6] Thus, group practices and groups of physicians with combined knowledge will be necessary to provide the most advanced care to patients.[1] Medicine is going through a revolution, transitioning from the old systems of the industrial age (resident education, hospital systems) where everyone was treated as a mass, to the 21st century, where each person and patient is recognized as unique. Treatments also will have to be individualized for each patient even more so than now as each person’s genetic code, when known, will allow physicians to design specific molecular treatments for that patient. Generalized treatments designed for the masses will disappear. In education, some are even looking at functional magnetic resonance imaging (fMRI) on each student and developing teaching models based on each person’s individual brain patterns and way of learning![18]

Artificial intelligence will provide physicians with decision choices from a large database of knowledge that no physician could ever remember or learn.[2,14] This huge source of medical information, which will be screened and updated constantly, will be at the fingertips of a physician by computer and Internet, providing the doctor with many statistically validated choices for the patient he/she is seeing in consultation. This possibility is not achievable by an individual physician, but groups of physicians come closer to this goal by exchange of knowledge. This new information will come from a huge worldwide database of continually expanding information on patients, which will surpass a physician’s past clinical experience that took a lifetime to develop and from which he/she drew clinical conclusions. Patient’s images will be captured automatically on the computer and will be compared with data bases of other patients’ images world wide to make a statistical diagnosis with alternative choices ranked by likelihood.[2,6] Radiologists will become less necessary for the traditional reading of images.[2] Much of this new technology will come from the interdisciplinary work of engineers, computer scientists, mathematicians and other specialists in different disciplines working together with physicians to solve medical and healthcare problems.[1,2,5,6]

In the past, the specialties of neurology, neurosurgery and psychiatry developed as different approaches to analyzing a variety of diseases affecting the brain. In the 21st century, these specialties will all become integrated as these clinicians discover that the genetic and cellular brain can manifest its behavior in a manner that will require the cooperation of all those specialists.[1,5,6] These specialty separations are a remnant of centuries before when doctors could only describe human behaviors but not understand the integrated brain behavior patterns that produce them. That was how descriptive science started at the time of the Renaissance. We also need the knowledge of those in education, social science, engineering, mathematics and other fields to better treat the patient and to enhance our own breadth of knowledge and understanding.[2] We are learning that the brain can grow new cells until the last breath of a person, and that neuroplasticity can overcome damage to the central nervous system (CNS) and allow an individual to continuously relate new information to old.[10] We are beginning to uncover the solutions to regeneration.[12,15]

The challenge for all societies around the world is “To Provide Quality Healthcare to the Largest Number of People at the Lowest Cost.”[2,6] This fundamental principle is true in the developed and in the developing world. How can such a goal be achieved? The answer to this question is by utilizing interdisciplinary medicine with the most efficient, cost-effective use of limited resources to provide the best care to all.

Yes, I do believe in liberty for the individual as opposed to government control, and that the Free Market is the most efficient way to provide medical care, but no country except the United States (USA) has come close to that goal.[4] Now, we, in the USA are becoming socialistic and that system will provide rationed, costly and inefficient care that will not be of good quality.[4,8] Yes, there are good systems in other countries but healthcare everywhere is bankrupting economies. Thus, we need to provide quality care for the largest number of people at the lowest cost.

Neuroscience in the future will be practiced in major centers where research can be conducted on the diseases common to that country or civilization. Neurosurgeons, neurologists, psychiatrists, rehabilitation physicians, neuroimgaing specialists, basic scientists from all fields and others will work in interdisciplinary teams to solve the disease challenges in these centers.[1,5,9] This arrangement will provide the most efficient use of the resources in a country. There may be more than one such center, but they should not be duplicative, if possible. They must work closely with the practicing physicians in all communities who provide the overwhelming majority of healthcare to the public.

We are beginning to learn that the central nervous system (CNS) is integrated with other organ systems in the body and is intimately involved in many diseases. We see myocardial changes in patients with Subarachnoid Hemorrhage (SAH), neurogenic pulmonary edema and gastrointestinal hemorrhage as diseases with a known relationship with the CNS. But psoriasis, ulcerative colitis, the immune system and even obesity, hypertension and diabetes all have nervous system components that will be revealed more as the century progresses.[13] Those facts mean even greater interdisciplinary relationships among physicians will be necessary. Genetic studies now show that epigenetic controls of the genome direct the expression of genes.[16] Environmental factors influence the epigenome and, thus, the behavior of the organism.[16] More of this information will become known as the century progresses. Even more interdisciplinary medicine will be required. Many neurological diseases will be molecular or genetically based and will require geneticists and other scientists to understand them. Many pediatric malformations are genetically based and will require multidisciplinary teams of scientists to understand and to treat those diseases, perhaps, in utero.

Yet, all this information and knowledge must be provided to the single patient or doctor far from the major medical center to enable each patient to have the most advanced care and the longest life possible.[2] That is what the public wants. That is what they work for and pay for. Technology will link those patients with their physician and the physician with the major centers.[2] The system will be integrated for the benefit of the patient, but costly duplication of services and technology should be avoided under the financial limitations of every country.

The structure of academic centers will change as these factors force physicians and academicians from the patterns of the past to change to adapt to the future. This experience will be convulsive for academic centers that are supposed to be open to change but are rigid and industrial in their thinking. I call it Academentia.[5,9] All over the world, countries have invested billions of dollars to build academic centers, but university departments still exist in the industrial age “silos” and do not communicate with each other. Incentives for interdisciplinary relationships need to be developed so that these huge resources of talent and facilities are combined to make the progress the people in each country need. Such a shift is a major change in the way academic centers think and operate.[5,9] From the government, to the academic center’s leadership, to the academicians, all need to have a vision of the future and a plan to achieve that goal or goals. Yet, as Margaret Thatcher stated, “Consensus is Mediocrity.”[3] Academic centers operate by consensus, not leadership and vision. Academic medical centers avoid the conflict produced by leaders to arrive at consensus from those who will not “rock the boat.”[3] The strategy of consensus governance is a failing strategy to achieve excellence and innovation in the 21st century. Change is coming rapidly, and we must be able to adapt to that change – not struggle with accepting it. Excellence needs to be encouraged and rewarded regardless of whether or not it differentiates some from others. That is life! The future of academic centers must be driven by a vision of the future and a plan to achieve that vision.

The rich and poor countries have followed Keynesian economic principles to financial bankruptcy.[7] You cannot spend your way out of debt. Therefore, countries all over the world are facing challenges because their failing economies were politically motivated to please a public (consensus) that would like everything for nothing. As yet, at the beginning of the 21st century, there are not enough resources in the world to provide everyone with everything. Therefore, fundamentally, we need to make choices. Will those choices be socialistic or free market based? Will the strategy for successful survival be based on individual incentive or government control and the support of an entitlement mentality? This is the political conflict we have seen in the last 100 years or more and even in previous civilizations.[11] Yet, a fundamental principle – learned by the generations who proceeded the accumulation of all the world wealth – continues to prevail to this day: “You should teach a person to fish, not give him/her a fish.” And yes, we do need to help those that cannot help themselves. This is basic human decency.

As the 21st century unfolds, futurists predict that there will be an abundance of food, water, shelter and even healthcare that we do not have now, resulting from scientific advances in many fields, which will solve the major problems facing civilizations around the world.[17] Artificial intelligence will have a huge impact on all aspects of society by providing thinking and creativity that will surpass what the human brain can do.[14] Today, we are all in the middle of a huge revolution in society, industry, politics, science and education. All of these segments of society are not changing at equal speeds.[17] How can an academic medical center and the practicing physician face this future is the question. Will they lead, or will they struggle against those who resist and are fearful of the change necessary to reach this inescapable goal of living successfully in the 21st century? They must work together and lead the society and governments into the future of medicine.

In the middle of all of this change, one thing that has not changed in generations of civilizations are the fundamental principles of life as taught by various religions and experience, including truth, honesty, hard work, common sense, good judgment, and the “golden rule of behavior” (Do unto others as you would have done unto you)–all of which are learned not from a computer, but from those with experience in life. These principles are fundamental to the success of any civilization, and will never change. Without those fundamental principles, civilization is doomed to self-destruction.

In the 21st century, everything will be very different, including medicine. The most difficult challenge is teaching people to “change.”[7] We are the first generation in human history to undergo, in one generation, a major revolution in all aspects of society. No other wave of change has swept civilization so rapidly.[17] But, as the Tofflers mention, another wave is coming in our lifetime, as we move into the space age to face outer space and civilizations previously unknown.[17] The 20th century is history. It is time to move into the 21st century.

## References

[1] Ausman, JI, Myers C, Church D (2004). Seven challenges for neurosurgeons and Physicians in the next Decade. Surg Neurol.

[2] Ausman JI (2006). A futurist look at healthcare. Surg Neurol.

[3] Ausman JI (2006). Leadership vs Consensus. Surg Neurol.

[4] Ausman JI (2007). Snapshot of the World-9/2007. Surg Neurol.

[5] Ausman JI (2002). Subspecialization according to Couldwell and Rovit. Surg Neurol.

[6] Ausman JI (2008). The Challenge for neurosurgery in the 21st Century. Surg Neurol.

[7] Ausman JI (2010). Welcome to the 21st Century. Surg Neurol Int.

[8] Ausman JI (2009). What is the Truth?. Surg Neurol.

[9] Ausman JI (2001). Why academic health centers are failing. Surg Neurol.

[10] Boakye M (2009). Implications of neuroplasticity for neurosurgeons. Surg Neurol.

[11] Durant W, Durant A (1997). The Lessons of History.

[12] Ieda M, Fu JD, Delgado-Olguin P, Vedantham V, Hayashi Y, Bruneau BG (2010). Direct Reprogramming of fibroblasts into functional cardiomyocytes by defined factors. Cell.

[13] Jannetta PJ, Fletcher LH, Grondziowski PM, Casey KF, Sekula RF (2010). Type 2 diabetes mellitus: A central nervous system etiology. Surg Neurol Int.

[14] Kurzweil R (2006). Reinventing humanity: The future of human- machine intelligence. Futurist.

[15] Pajcini KV, Corbel SY, Sage J, Pomerantz JH, Blau HM (2010). Transient inactivation of Rb and ARF yields regenerative cells from postmitotic mammalian muscle. Cell Stem Cell.

[16] Solaroglu I, Zhang J (2009). The neurosurgical role of epigenome. Surg Neurol.

[17] Toffler A, Toffler H (2006). Revolutionary Wealth.

[18] Tucker P (2007). The Rise of brain focused teaching. Futurist.

